# Modulating TTA efficiency through control of high energy triplet states†

**DOI:** 10.1039/d1tc05292f

**Published:** 2022-02-22

**Authors:** Andrew J. Carrod, Alexei Cravcenco, Chen Ye, Karl Börjesson

**Affiliations:** Department of Chemistry and Molecular Biology, University of Gothenburg Gothenburg 41296 Sweden karl.borjesson@gu.se; Department of Chemistry, Uppsala University Uppsala 752 36 Sweden

## Abstract

An ideal annihilator in triplet–triplet annihilation photon upconversion (TTA-UC) can achieve a maximum of 50% quantum efficiency. This spin statistical limit depends on the energies of the triplet states of the annihilator molecule, with only 20% quantum efficiencies possible in less-optimal energy configurations (*E*_T_2__ ≤ 2*E*_T_1__). Our work utilises three perylene analogues substituted with phenyl in sequential positions. When substituted in the bay position the isomer displays drastically lowered upconversion yields, which can be explained by the system going from an ideal to less-ideal energy configuration. We further concluded position 2 is the best site when functionalising perylene without a wish to affect its photophysics, thus demonstrating how molecular design can influence upconversion quantum efficiencies by controlling the energetics of triplet states through substitution. This will in turn help in the design of molecules that maximise upconversion efficiencies for materials applications.

## Abstract 2

It is shown here that positional isomerism of perylene substitution affects high energy triplet states differently. This in turn influences the quantum efficiency of triplet–triplet annihilation photon upconversion.

## Graphical abstract

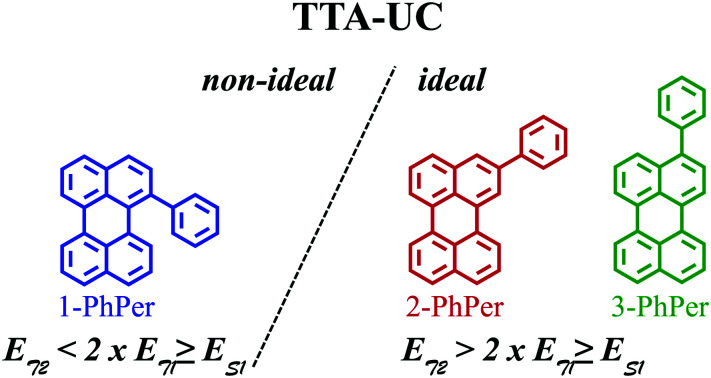

## Introduction

Triplet–triplet annihilation upconversion (TTA-UC) is a process that converts two low energy photons to one of higher energy.^1–3^ Environmental and economic benefits of maximising upconversion quantum yields are evident, as TTA-UC has been identified as a method to increase solar cell efficiencies beyond that of the Shockley–Queisser limit.^4–6^ Furthermore, TTA-UC has been successfully demonstrated in many photovoltaic,^6–16^ photocatalytic,^17–19^ and biosensing^20–22^ applications, often where achieving maximum upconversion quantum yield (QY) is a figure of merit. A second figure of merit is the threshold intensity (*I*_th_). The *I*_th_ indicates the point at which TTA becomes the dominant mechanism of triplet state elimination. It should ideally lie below the power of the solar spectrum integrated over the absorption band of the upconversion system, for it to be relevant for photovoltaic applications.

In order to maximise the upconversion efficiency, it is important to have a clear understanding of which factors drive the process. Mechanistically, TTA-UC utilises a sensitiser and an annihilator. The sensitiser absorbs a photon at low energies and transfers the energy to the annihilator through triplet energy transfer (TET).^23^ Two diffusing annihilator molecules subsequently come into close contact, and the triplet states recombine through triplet–triplet annihilation,^24^ promoting one annihilator molecule to a higher energy excited state and the other to its ground state. Emission then occurs as it would by direct excitation of the singlet state (fluorescence). The most important prerequisite for an annihilator is that twice the energy of the first excited triplet state must equal or exceed that of the first excited singlet state (2*E*_T_1__ ≥ *E*_S_1__).^25^ Furthermore, for the highest possible upconversion quantum yields, the energy of the second triplet state should lie above twice the first (*E*_T_2__ > 2*E*_T_1__ ≥ *E*_S_1__).^26–28^ This prerequisite is not met by some of the most popularly used annihilators, therefore limiting the efficiency of for instance diphenylanthracene.^29^ Clearly then, the ability to control the *E*_T_2__ whilst minimally affecting the *E*_S_1__ and *E*_T_1__ energies would be a boon for the TTA-UC field.

Perylene has been widely studied as an annihilator in TTA systems, as it has near unity fluorescence QY, visible emission wavelengths and S_1_ and T_1_ state energies of around 2.7 and 1.5 eV, respectively.^30–34^ Perylene is further advantageous, as the T_2_ energy is higher than twice the T_1_ energy, allowing for highest possible upconversion yields. Moreover, its planar polyaromatic structure allows for substitution at multiple positions. The substitution position on the perylene ring will likely alter the photophysical properties, and may render the system more or less suitable for TTA-UC applications. Perylene substitution has been previously explored in relation to solubility and mechanistic studies of aggregation.^35–38^ Previous studies have investigated a library of substituted perylenes computationally and experimentally, with consideration given to their excited state energetics with differing functional groups, or multiple substitutions.^39^ However, no systematic investigation has been carried out on the modulation of perylene photophysics with site selective substitution. It is therefore important to provide an understanding of the influence of substitution position, especially on *E*_T_2__, *E*_T_1__, and *E*_S_1__.

Herein, we investigate the substituent position-excited state energetics relationships, using a series of phenyl substituted perylenes. We also detail the TTA-UC properties of the perylene derivatives, and use computational methods to justify the differences observed in the experimentally obtained spectra. We observed that when the theoretical energetic requirement for high-efficiency TTA-UC (*E*_T2_ > 2*E*_T1_) no longer holds true, a significantly lower value is also achieved experimentally. We furthermore give an explicit recommendation on where to substitute perylene in order to affect its photophysics as little as possible. This work will in turn be useful in the design of perylene derivatives for both traditional fluorescence and TTA-UC applications. Furthermore we show the possibility to modify *E*_T2_ through substitution in order to go between ideal and less-ideal energetic regimes.

## Results and discussion

### Design, synthesis and structure of perylene derivatives

**Scheme 1:**
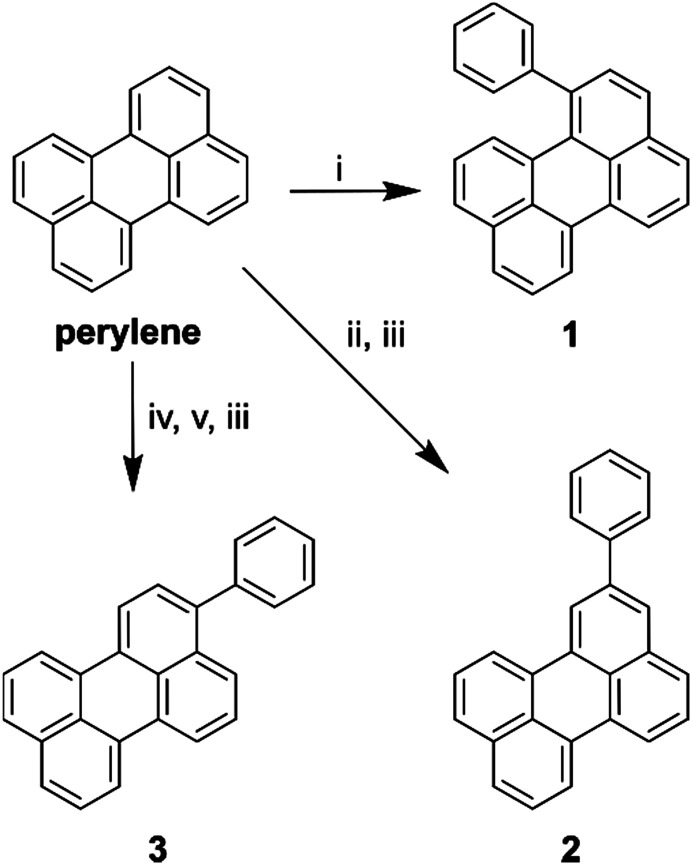
Synthetic routes to reach each perylene derivative where (i) PhLi, THF, −78 °C; (ii) B_2_Pin_2_, dtbpy, [Ir(COD)_2_(OMe)_2_], THF, 80 °C; (iii) PhBr, Pd_2_(dba)_3_, RuPhos, K_2_CO_3_, PhMe, 100 °C; (iv) NBS, THF, RT; (v) B_2_Pin_2_, KOAc, Pd(dppf)_2_Cl_2_, 1,4-dioxane, 70 °C.

In order to investigate the effect of positional isomerism on the photophysics of perylene derivatives, we synthesised phenyl substituted perylenes at positions 1, 2, and 3 (Scheme 1). Phenyl substituents have a near zero, and equal Hammett value in any measurable position.^40,41^ Consequently, any observed effects in the luminescence properties are postulated to arise solely from a change in conjugation length, and not from electron inductive or withdrawing effects. Arylation of the perylene core was either achieved by the direct use of phenyllithium in the case of 1-phenylperylene, or Suzuki couplings in the case of 2-, and 3-phenylperylene (Scheme 1, see ESI† for details of synthesis).

### Photophysics of perylene derivatives

**Fig. 1:**
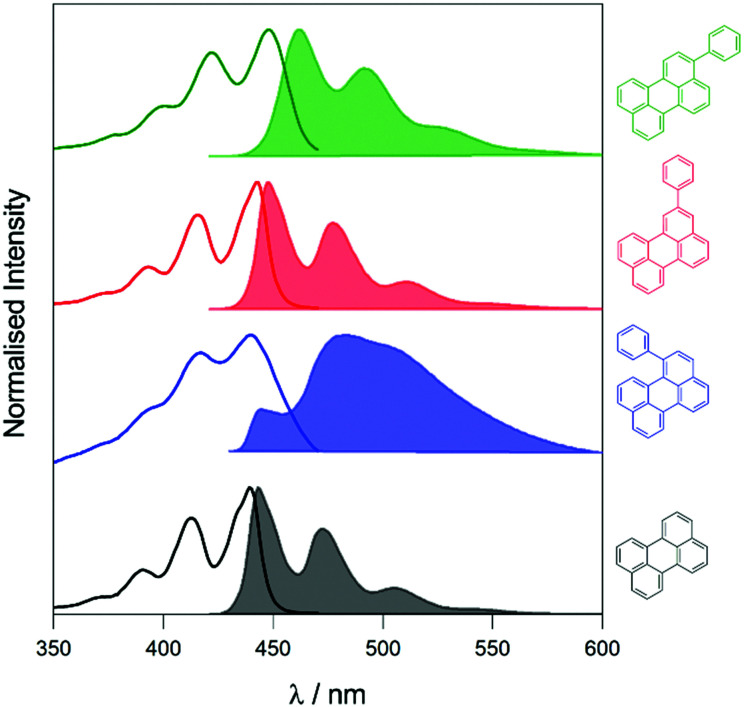
UV-Visible absorption (solid line) and emission (solid filled, *λ*_exc_ = 410 nm, [perylenes] = 1–2 μM) spectra of perylene derivatives in toluene. Structures of the compounds are displayed in the colour corresponding to their spectra.

To elucidate the effect of the phenyl substituents on the singlet-state photophysics of all derivatives, we collected electronic absorption and fluorescence spectra for each compound in toluene. The spectra are shown in Fig. 1 with further data of merit summarised in Table 1. It is observed from the UV-Vis absorption spectra that there is a bathochromic shift in the absorption maxima of 2, 5 and 10 nm (104, 258 and 510 cm^−1^) compared to that of naked perylene, for 1, 2, and 3, respectively. The fluorescence maxima of 2 and 3 show a mirror image relationship of the emission with the UV-Vis as expected. Both 2 and 3 also show characteristically high molar extinction coefficients, and high emission quantum yields. The case of 1 is intriguing however, with lower molar extinction coefficient and fluorescence quantum yield observed. To understand this we calculated the radiative and non-radiative rate constants for each derivative. For 2 and 3, values are comparable to that of perylene, with a slight increase in the non-radiative rate for 2 consummate with the reduction in fluorescence quantum yield.

**Table 1:** Experimentally determined photophysical properties of perylene derivatives

Compound	*λ*_max_/nm (*ε*/10^4^ M^−1^ cm^−1^)	*λ*_em_ a/nm	*Φ* b/%	*τ* c/ns	*k*_r_/10^8^ s^−1^	*k*_nr_/10^7^ s^−1^
Perylene	438 (3.9)	443	99	3.8	2.6	0.3
1	440 (1.8)	482	70	3.9	1.8	7.7
2	443 (3.6)	447	93	3.9	2.4	1.8
3	448 (3.7)	462	97	3.3	2.9	0.9

a Values are determined in toluene with *λ*_exc_ = 410 nm, the value for the most intense peak is given.

b Emission quantum yield. Values are referenced using the reported value of perylene in benzene.

c Luminescence lifetime. Values are determined in toluene at 375 nm.

For 1, whilst the radiative rate decreases, we also observed a large increase in the non-radiative rate constant, which explains the large discrepancy in the fluorescence quantum yield. The absorption and emission spectrum for 1 also show a non-mirror image vibronic structure. The ratio of intensities of the vibronic progression differ considerably. The intensity of the vibrational progression depends on the Hung-Rhys factors, indicating a considerable translation of the excited state potential from the Frank-Condon state to the geometry relaxed excited state. This has been observed for other organic fluorophores.^42^

### TTA-UC of perylene derivatives

**Fig. 2:**
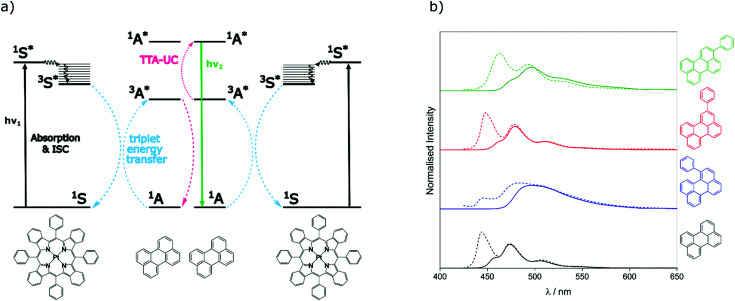
(a) Energy-transfer scheme involved in the TTA-UC process, with each constituent energy transfer process coloured and labelled. The structures shown below are that of the PtTBTP sensitiser and naked perylene annihilator used in this work. (b) Emission spectra collected (solid lines) and spectra from Fig. 1 normalised (dashed) based on the intensities of the unfiltered peaks of perylene derivatives in toluene (*λ*_exc_ = 617 nm, [annihilator] = 1 mM, [sensitiser] = 6 μM. 0.85 mJ).

To assess the TTA-UC properties shown by the perylene derivatives we used the triplet sensitiser platinum tetra-benzo–tetra-phenyl–porphyrin (PtTBTP, Fig. 2a). This sensitiser has a long triplet excited state lifetime and is known to be able to sensitise perylene.^31,32,43^ The absorption and emission spectra of the sensitiser collected in toluene are given in Fig. S1 (ESI†). Emission spectra after sensitiser excitation were collected in toluene solution using 6 μM PtTBTP in combination with each of the presented perylene derivatives, at a concentration of 1 mM. Excitation with a pulsed laser (617 nm) leads to strong emission in the range 450 nm to 540 nm due to TTA (Fig. 2b), with the excitation power density of the laser being much higher than the measured threshold intensities (Fig. S2, ESI†). We observe that the emission energies match well with those observed in the directly excited singlet state, only with the first vibrational peak undetectable due to the large inner filter effect. The upconversion quantum yield (*Φ*_UC_) of each system was measured, and is reported in Table 2. *Φ*_UC_ observed for perylene is in agreement with previously reported values.^32,35^ Compounds 2 and 3 are slightly lower, whilst 1 shows a far reduced *Φ*_UC_.

**Table 2:** Parameters of perylene derivatives for TTA-UC

Compound	*Φ*_uc_/%	*Φ*_smax_/%	*K*_TET_/10^9^ M^−1^ s^−1^	*β*	*K*_t_/10^3^ s^−1^	*K*_TTA_/10^9^ M^−1^ s^−1^
Perylene	3.2	5.0	2.0	0.54	3.2	1.9
1	1	2.2	2.5	0.66	1.4	1.4
2	2.6	4.4	2.2	0.41	4.8	1.7
3	2.3	4.4	2.0	0.54	2.0	1.2

To assess the reason behind this lower TTA-UC quantum yield, it is first pertinent to consider the definition of *Φ*_UC_.^3^ This parameter is defined in accordance with eqn (1). Where *Φ*_sing_ is the yield of generated emitted photons, and *Φ*_exp_ is the outcoupling efficiency.

1 *Φ*_UC_ = *Φ*_sing_*Φ*_exp_

To account for outcoupling losses due to annihilator and sensitiser reabsorption, we normalise the fluorescence spectra taken at low concentrations (Fig. 1) to the intensities observed for the unfiltered 0–2 vibrational peaks in Fig. 2. We can then calculate for each derivative the losses brought about by high annihilator and sensitizer concentrations. In this way, we obtain *Φ*_sing_, the yield of photons emitted through TTA-UC. However, in the context of our research question, the outcoupling efficiency only accounts for inner filter effects. To fairly compare the efficiencies of singlet generation in the perylene derivatives, we must further account for the fluorescence quantum yield of each compound. We can do this by considering eqn (2). Where *Φ*_f_ accounts for the fluorescence quantum yields of the perylene derivatives.

2 *Φ*_sing_ = *Φ*_smax_*Φ*_f_

Division of *Φ*_sing_ by *Φ*_f_ therefore gave us the value of *Φ*_smax_, that is the maximum yield of singlet states obtained by TTA-UC (Table 2). The *Φ*_smax_ values for 2 and 3 are within error of the value calculated for naked perylene. However, compound 1 still displayed a much reduced *Φ*_smax_ value compared to perylene and therefore further investigation of this disparity was warranted.

So far, we have adjusted the upconversion efficiency for processes happening after the annihilation step. We will now assess efficiencies in all stages within the UC process to understand the continued difference between the derivatives. Firstly we investigated the rate of triplet energy transfer from the sensitiser to each annihilator (*k*_TET_) using the quenching of the sensitiser emission (Fig. S3, ESI†). Derived *k*_TET_ values of the derivatives based on Stern–Volmer kinetics did not significantly deviate from those obtained for perylene in toluene,^31,44,45^ and are near diffusion limited processes. The *Φ*_TET_ was >99% for all experiments (Fig. S3b, ESI†). Secondly, we assessed the rate of intrinsic triplet decay and of triplet–triplet annihilation, both of which contribute the loss of annihilator triplet-excited states. We fitted the time-resolved decay of the UC emission (Fig. S4, ESI†) in the same manner as previous works using known equations (eqn (3)) to obtain values for the intrinsic triplet decay (*k*_t_).^46^3
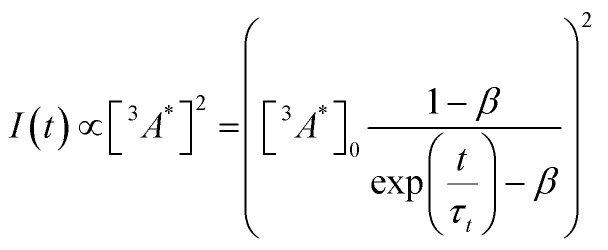
where *I*(*t*) is the upconverted emission intensity, *β* is a dimensionless parameter between 0 and 1 expressing the initial decay fraction resulting from second-order channels, *t* is time, and *τ*_*t*_ is the triplet excited state lifetime. The analytical solution to the term *β* is known,^24,46,47^ and to calculate *k*_TTA_ requires only the initial concentration of triplet annihilators to be known (eqn (4)). We used the intensity of the T_1_–T_*n*_ transition of perylene (*ε*_T_1__485 nm_ = 13 400 M^−1^ cm^−1^) to calculate [^3^A*]_0_ in the upconversion solution.^48^ As there is no significant deviation in the Stern–Volmer kinetics for each derivative, the value of [^3^A*]_0_ can be assumed to be equal for all annihilators, in order to obtain the rate constant for TTA (*k*_TTA_, Table 2).4
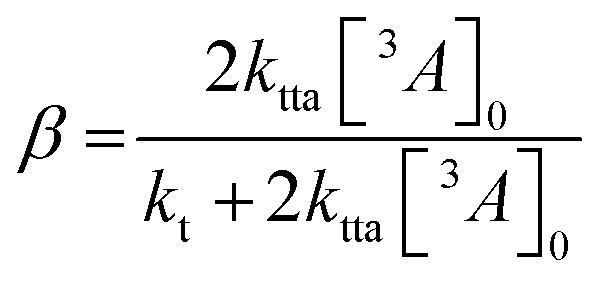


No major discrepancies in the values for *k*_*t*_, *β* or *k*_TTA_ were found (Table 2), in line with what could be expected from previous works.^35^ In combination these results rather puzzlingly indicate that all steps within the TTA-UC process are occurring rapidly. In light of the definition of *Φ*_smax_ (eqn (5)), and knowing that intersystem crossing (ISC) and TET are equal in all cases, we considered *Φ*_TTA_ to be the difference.

5 *Φ*_smax_ = *Φ*_ISC_*Φ*_TET_*Φ*_TTA_

The value of *Φ*_TTA_ is limited by the spin statistical probability of forming a singlet state upon the annihilation event (*η*_TTA_). Spin statistics have been well defined in the TTA-UC literature with maximum *η*_TTA_ values of 1 or 0.4. Maximum *Φ*_TTA_ values are therefore either 0.5 or 0.2 owing to the generation of one photon from two incident photons.^26,28^ This is determined by whether the ideal (*E*_T_2__ > 2 × *E*_T_1__ ≥ *E*_S_1__) or non-ideal (*E*_T_2__ < 2 × *E*_T_1__ ≥ *E*_S_1__) conditions are met by the annihilator. It is noteworthy to indicate excimer formation was not observed in perylene or any of the derivatives studied. Perylene is much less prone to excimer formation in toluene than in for instance THF.^32,35^ Emission arises from monomeric species, and energy is not lost to excimer formation, consequently it is only necessary to investigate the triplet state energies of the molecules themselves.

### TD-DFT and transition dipole density calculations

**Fig. 3:**
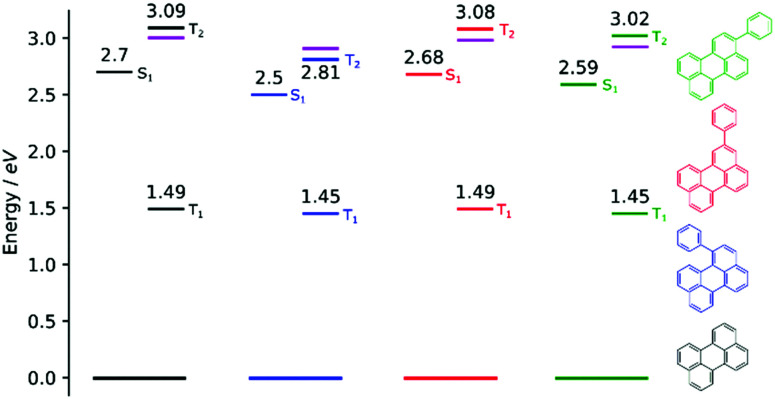
Energy level diagram with calculated values of S_1_, T_1_ and T_2_ energy levels for the compounds used in this study. A purple line indicates the energy of 2×T_1_ for each compound. The energy levels here are based on the triplet ground state geometry, reflecting their relevant energies in the TTA regime.

In order to investigate if the energetics of the excited states in the perylene analogues are responsible for the differing *Φ*_TTA_, we used density functional theory (DFT) as well as time-dependent DFT (TD-DFT) in a similar way to that previously described.^49^ Our TD-DFT calculations were carried out for each molecule, elucidating S_1_, T_1_ and T_2_ energies. These results are summarised in Fig. 3 and further details can be found in the ESI.† The calculated energies for perylene in all states are very close to that previously reported.^31,49,50^ The T_1_ state energies are similar in all phenyl substituted compounds, with values of 1.45, 1.49 and 1.45 eV for 1, 2 and 3, respectively. A large difference is evident in the T_2_ energies, where the values for all compounds except 1 are above 3.0 eV satisfying the ideal conditions for TTA molecules (*E*_T_2__ > 2 × *E*_T_1__ ≥ *E*_S_1__). A large drop to 2.8 eV is observed for the T_2_ energy for 1, meaning that this compound is non-ideal for TTA (*E*_T_2__ < 2 × *E*_T_1__ ≥ *E*_S_1__). The lowering of the energy gap between the first and higher triplet states in 1 relative to perylene is experimentally supported by transient absorption spectroscopy. The T_1_–T_*n*_ transition observed in 1 lowers in energy by 0.15 eV compared to perylene (Fig. S5, ESI†). This indicates that there is a room for tweaking the energetics of small organic molecules in order to go between ideal and non-ideal TTA energetics. The outlook being the possibility to take an energetically non-ideal annihilator and turn it into an ideal one through substitution.

**Fig. 4:**
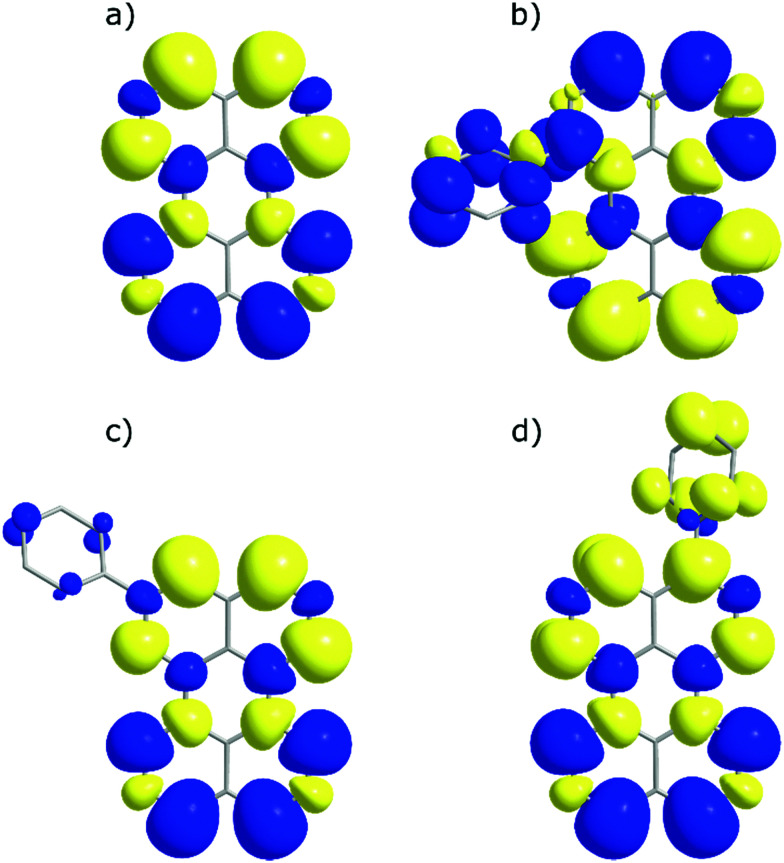
Transition dipole moment density maps for (a) perylene, (b) 1, (c) 2, and (d) 3. The size of the clouds relates to the magnitude of the density. Excited state transitions are calculated based upon a singlet ground state.

To look closer at the effect of the substitution pattern on the S_1_ state, we further utilised the DFT results to yield transition dipole moment densities. The transition dipole moment densities for the S_0_ → S_1_ state were calculated using a multifunctional wavefunction analyser.^51–53^ Graphical representations of the transition dipole moment densities for all compounds are given in Fig. 4. The results clearly demonstrate a position dependent influence of substitutions on the perylene excited state transition. The largest bathochromic shift is shown by 3, which can be explained by an elongation of the transition dipole moment. The phenyl substituent of 2 contain very little transition dipole density. This derivative also show the largest similarity with perylene when it comes to absorption and emission envelopes and Stokes shift. The phenyl substitution of 1 has a considerable transition dipole moment density, and the direction of the transition dipole moment is therefore significantly changed from perylene. However, since also the substitution induces a twist in the aromatic system (the bay area show a dihedral angle of 15 degrees), it is difficult to disentangle substitution *vs.* twisting effects on the photophysics. To summarise, when derivatising perylene, position 2 is preferable if minimal change to the photophysics is wanted.

## Conclusion

In summary, three phenylperylene derivatives were synthesised (1–3) with positional isomerism. We found significant deviations in the fluorescence properties of 1 from those of 2, 3 and naked perylene. The most unaffected derivative regarding photophysics, was that of 2, therefore we concluded this to be the best position when functionalising perylene without a wish to affect its photophysics. Regarding TTA-UC, 2 and 3 showed unremarkable differences in their upconversion quantum yields, however 1 showed a significant difference. Calculations of the T_2_ state energies for each derivative offered an insight to the reason behind this drop. With functionalisation in the bay position of perylene, we observed a significant lowering of the second triplet excited state energy. Synthetic modifications can thus lead to selective modulation of the second triplet state energy. This in turn can convert an energetically non-ideal TTA-UC annihilator into an ideal annihilator or vice-versa. This is beneficial for the optimisation of any TTA-UC material in which the largest upconversion quantum yield is desired.

## Author contributions

Photophysical studies were carried out by AJC, with support from CY. Synthesis was carried out by AC. Computational studies were carried out by AJC and AC. KB conceived and coordinated the work. AJC and KB drafted the manuscript and all authors discussed the results, commented on the manuscript and approved the final version of the manuscript.

## Conflicts of interest

There are no conflicts to declare.

## Supplementary Material

TC-010-D1TC05292F-s001
