# Supplementary material for: Modulating TTA efficiency through control of high energy triplet states
Source: J Mater Chem C Mater. 2022 Feb 22;10(12):4923–8. doi: 10.1039/d1tc05292f (PMC8944256; doi:10.1039/d1tc05292f)
Supplement: TC-010-D1TC05292F-s001 [file TC-010-D1TC05292F-s001.pdf]

Electronic Supplementary Material (ESI) for Journal of Materials Chemistry C.
This journal is © The Royal Society of Chemistry 2022

Supplementary Information for: Modulating TTA efficiency through control of high energy triplet states

Andrew J. Carrod, Alexei Cravcenco, Chen Ye and Karl Börjesson*

Email: karl.borjesson@gu.se

Table of Contents

Methods and materials 2

1. **Sensitiser photophysics** 3
2. **I_{th} values** 3
3. **Triplet energy transfer rate** 4
4. **Triplet lifetime** 5
5. **Transient absorption** 6
6. **Synthesis** 7
   a) 1-phenylperylene 7
   b) 2-phenylperylene 8
   c) 3-phenylperylene 9
7. **NMR Spectra of final compounds** 10
8. **References** 13

Methods and materials

Synthesis: All starting materials were purchased from Sigma-Aldrich Chemical Co. and used without further purification unless otherwise stated. All moisture- and oxygen-sensitive reactions were carried out using Schlenk techniques in oven-dried glassware. Solvents used for moisture- and oxygen-sensitive reactions were dried using an MBraun MB SPS-800 solvent purification system. Preparative TLC was performed on AnalTech silica gel coated glass plates (Sigma-Aldrich, 2000 µm). ^{1}H NMR (nuclear magnetic resonance) spectra were recorded on a Varian spectrometer at 400 MHz where ^{13}C NMR spectra were recorded at 101 MHz. Chemical shifts are given in parts per million using tetramethylsilane as an internal standard. Mass spectrometry data was obtained using a 5977E GC/MSD from Agilent. High-resolution MS was obtained from an Agilent 1290 infinity LC system equipped with an autoSampler in tandem with an Agilent 6520 Accurate Mass Q-TOF LC/MS.

Photophysics: All TTA-UC samples were prepared in an Mbraun glove box having oxygen and water levels less than 1 ppm. Cuvettes were sealed with cap and PTFE septum. Photon upconversion measurements were performed immediately after the preparation. Steady state emission of TTA-UC samples with PtTBTP was measured on an Edinburgh Instruments FLS 1000 spectrofluorometer. A Light Emitting Diode (LED) with collimator and focus lenses was used as a non-coherent excitation source. The LED light source (617 nm, Thorlabs M617L3 mounted LED), power supply and all the optical units were purchased from Thorlabs, Inc. The photoluminescence quantum yield of the luminescent species were determined from an indirect method with standard reference as recommend by IUPAC.[1, 2] The quantum yield of the testing samples were calculated by the following equation S1:

$$\Phi_x^i = \frac{F^i f_s n_i^2}{F^s f_i n_s^2} \Phi_f^s \qquad \text{(Equation S1)}$$

Where Φ_x^i is the quantum yield of either upconversion or fluorescence. Φ_f^s is the known quantum yield of the standard. F^i and F^s are the integrated areas of the sample and standard emission spectra, respectively. f_i and f_s are the absorbance for the sample and reference, respectively. n_i and n_s are the refractive indices of the sample and reference solution, respectively. The excitation source (and wavelength of which) and detector was kept constant between reference and sample, therefore flux can be considered equal and ignored. Standard reference materials were selected by the excitation and emission spectrum range according to IUPAC recommendations, with perylene in benzene ($\Phi_f = 0.99$) used as a reference for Φ_f^i and cresyl violet in methanol ($\Phi_f = 0.57$) used as a reference for Φ_{UC}^i.[2]

Prompt fluorescence decay was recorded by time-correlated single photon counting (TCSPC) on an Edinburgh FLS 1000 spectrofluorometer with a pulsed diode laser (375 nm, 1 MHz) as the excitation source and a MCP-PMT as detector.

Transient absorption was measured on an Edinburgh Instrument LP 980 spectrometer, with a Spectra-Physics Nd:YAG laser (617 nm, pulse width ~7 ns) coupled to a Spectra-Physics primoscan optical parametric oscillator (OPO) as excitation. PMT (Hamamatsu R928) or image intensified CCD camera (ICCD, Andor DH320T-25F-03) detectors were used for recording transient kinetics or spectra, respectively.

Optimized ground-state energies and vibrational frequencies were calculated using density-functional theory (DFT), adiabatic and vertical excitation energies, with time-dependent DFT (TD-DFT), using the B3LYP/LANL2DZ level. All calculations were done with the Gaussian 16 program package.

1. Sensitiser photophysics

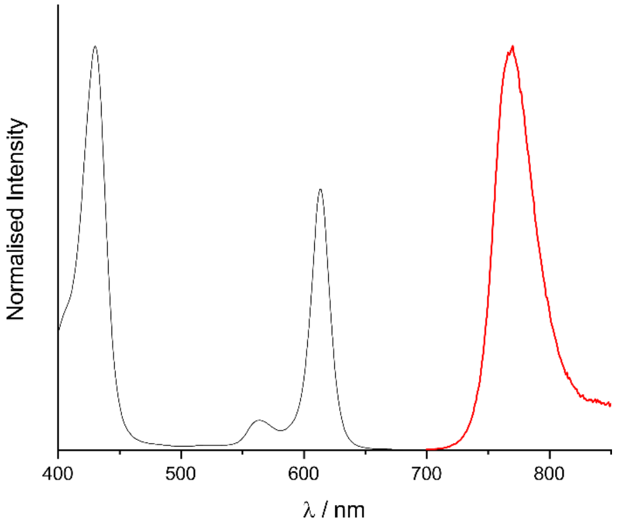

Figure S1: UV-Visible absorption (black line) and emission (red line, λ_{exc} = 613 nm) spectra of PtTBTP in toluene.

2. I_{th} values

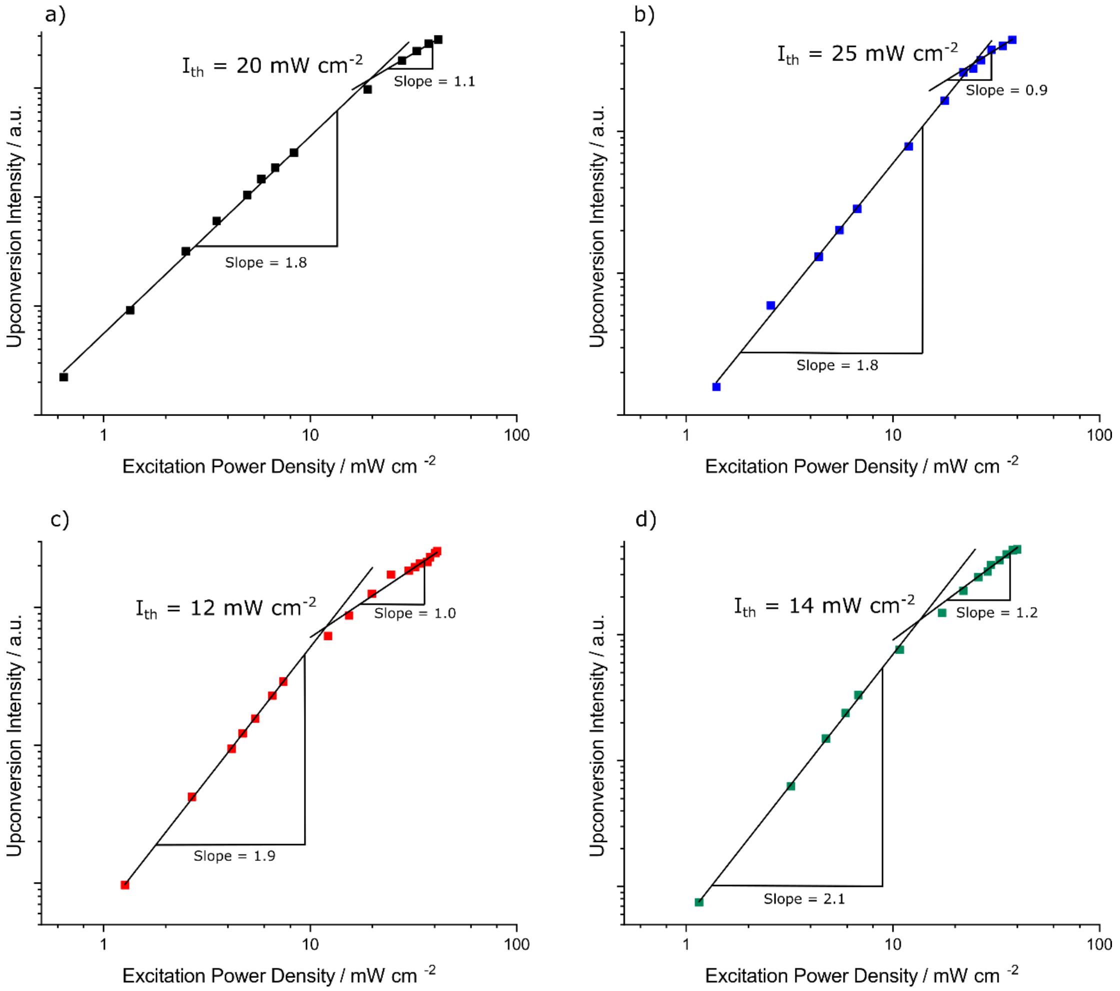

Figure S2: Double logarithmic plots of upconversion intensity versus the excitation power density for solutions of 6 μM PtTBTP and 1 mM of (a) perylene, (b) **1**, (c) **2**, (d) **3** in toluene.

3. Triplet energy transfer rate

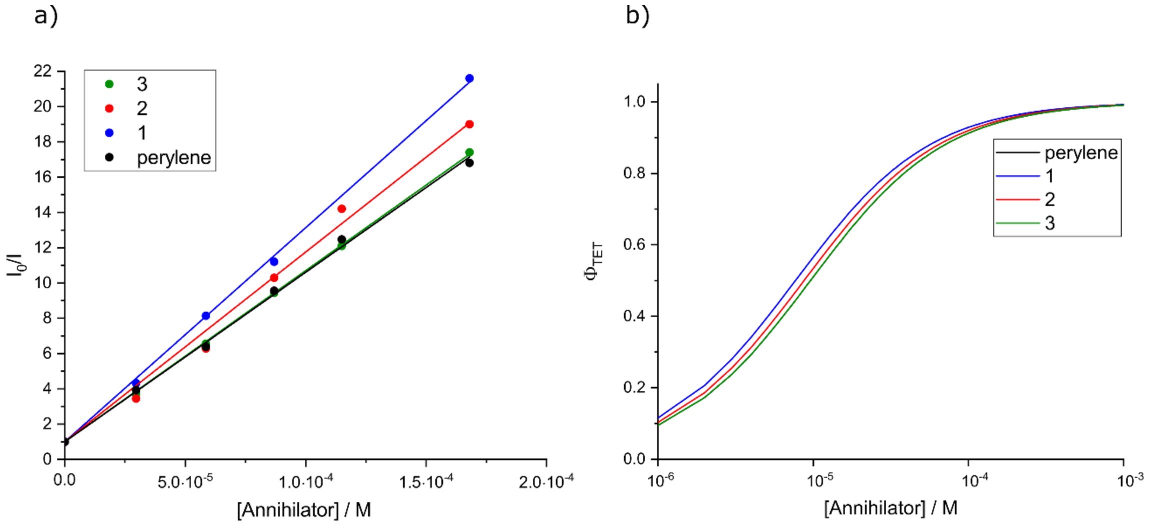

Figure S3: (a) Stern-Volmer plot indicating quenching as a function of annihilator concentration. (b) Figure showing how the Φ_{TET} differs with the concentration of annihilator. Values are fit using Equations S2 and S3.

The TET from the sensitiser involves diffusion and energy transfer. The energy transfer kinetics are therefore dynamic, parameters can be plotted (Figure S3) and a value of k_{TET} calculated based on the Stern-Volmer relationship given below in Equation S2:

$$\frac{\tau_0}{\tau} = \frac{I_0}{I} = 1 + k_{TET}\tau_0[A] \qquad \text{(Equation S2)}$$

This is further adapted into Equation S3 to give a value of the energy transfer efficiency Φ_{TET}, which is also shown in Figure S3.

$$\Phi_{TET} = \frac{k_{TET}\tau_0[A]}{1+k_{TET}\tau_0[A]} \qquad \text{(Equation S3)}$$

4. Triplet lifetime

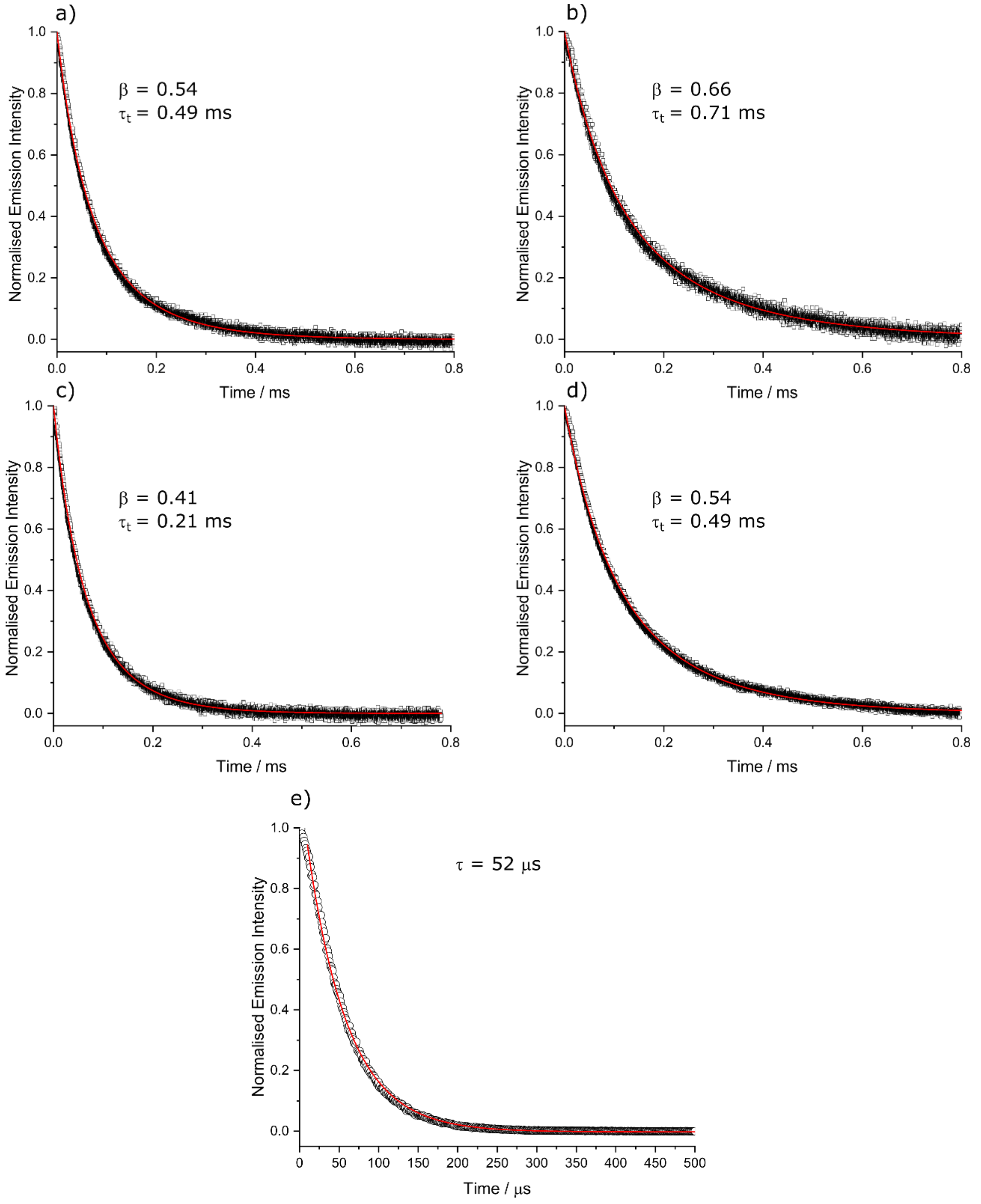

Figure S4: Upconverted emission intensity plots after pulsed laser excitation at 617 nm for (a) perylene, (b) **1**, (c) **2**, (d) **3**. Plots are fitted using equation S3 where n=2. [sensitiser] = 6 μM, [annihilator] = 1 mM. (e) Time resolved emission intensity plot of the sensitiser PtTBTP in toluene.

5. Transient absorption

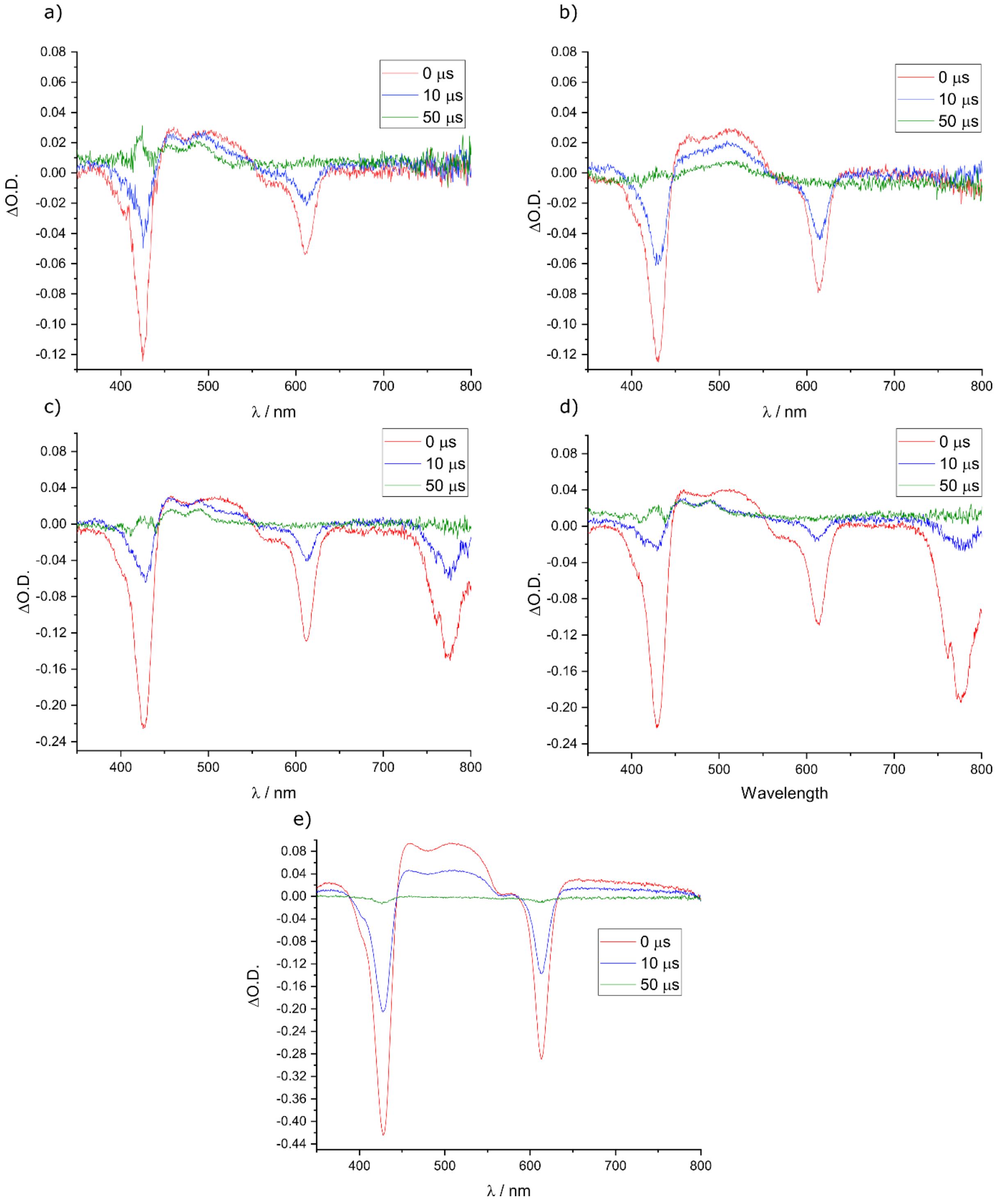

Figure S5: Transient absorption spectra of solutions of 6 μM PtTBTP and 1 mM of a) perylene, b) **1**, c) **2**, d) **3** in toluene. e) spectra of 6 μM PtTBTP in toluene. All spectra were collected at probe time delays of 0 μs, 10 μs and 50 μs. The residual peak at 50 μs relates to the T_1 to T_n absorbance of the perylene species as there is no remnant signal from the sensitiser as observed in e).

6. Synthesis

a) 1-phenylperylene

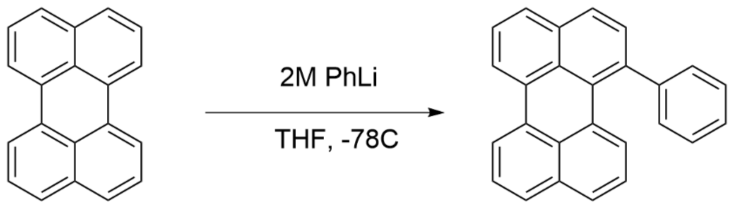

Scheme S1: Synthetic route to 1-phenylperylene

1-phenylperylene: Perylene (252 mg, 1 mmol) was dissolved in THF (10 mL) and cooled to -78 °C. A solution of PhLi (0.58 mL, 1.9 M, 1.1 mmol) was added dropwise under argon. The mixture was stirred for 45 min at -78°C, warmed to 0 °C for 1 h, after which the reaction mixture was allowed to reach room temperature. After stirring at room temperature for one hour the solution was again cooled to -78 °C, followed by the addition of a solution of I_2 (200 mg, 0.8 mmol) in THF (2 mL). After warming to r.t. over 25 min, dichloromethane (50 mL) and saturated aqueous $Na_2S_2O_3$ (50 mL) were added and the organic phase was separated and dried over Na_2SO_4 before filtering. Solvent was removed under reduced pressure and the solid residue was purified by preparative TLC in cyclohexane. dichloromethane was used to remove the product from the relevant collected silica. The solvent was evaporated to afford 1-phenylperylene as a dark yellow powder. Combined fractions yielded (112 mg, 34%). mp = 130 °C (degradation). λ_{max} / nm (ε / 10^4 M^{-1} cm^{-1}) 440 (1.8), 415 (1.6), 395 (0.8). ν_{max}/cm^{-1} = 3053 (CH). ^1H NMR (400 MHz, $CDCl_3$) δ = 8.20 – 8.15 (m, 2H), 7.73 – 7.64 (m, 3H), 7.55 – 7.32 (m, 10H), 6.97 (t, *J* = 7.9 Hz, 1H). ^{13}C NMR (101 MHz, $CDCl_3$) δ = 145.2, 138.3, 134.1, 133.6, 131.6, , 131.1, 130.1, 130.0, 129.5, 129.0, 128.8, 127.8, 127.7, 127.5, 127.1, 127.1, 127.0, 126.5, 126.3, 126.2, 125.5, 121.0, 120.2, 120.1. GC-MS (ES^+): $(M)^+$ 328.2

b) 2-phenylperylene

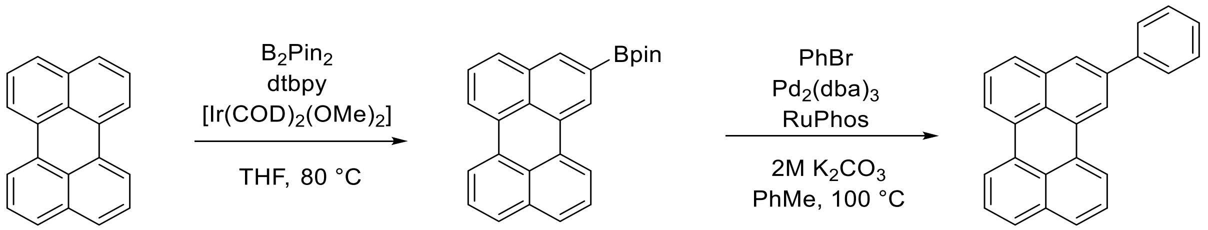

Scheme S2: Synthetic route to 2-phenylperylene

4,4,5,5-tetramethyl-2-(perylene-2-yl)-1,3,2-dioxaborolane: In a glove box, perylene (200 mg, 0.79 mmol), B_2Pin_2 (221 mg, 0.87 mmol), $[Ir(COD)_2(OMe)_2]$ (52 mg, 0.079 mmol) and dtbpy (42 mg, 0.079 mmol) were placed in a microwave vial and capped with a Teflon lined cap. The vial was taken out of the glove box and dry THF (15 mL) was added via a syringe under argon. The reaction mixture was stirred at 80 °C for 24 h, where after it was allowed to reach ambient temperature. Solvent was removed under reduced pressure and the crude residue was purified by silica gel flash chromatography (isocratic toluene elution). The title pinacol ester was obtained as an orange powder (185 mg, 62%). ^{1}H NMR (400 MHz, $CDCl_3$): δ = 8.55 (d, J = 0.9 Hz, 1H), 8.32 (dd, J = 7.6, 1.0 Hz, 1H), 8.22–8.14 (m, 3H), 7.73–7.70 (m, 1H), 7.68–7.65 (m, 2H), 7.50–7.43 (m, 3H), 1.42 (s, 12H). ^{13}C NMR (101 MHz, $CDCl_3$): δ = 135.9, 134.7, 134.0, 131.3, 131.2, 131.2, 130.3, 130.3, 128.8, 128.5, 127.8, 127.8, 127.7, 126.5, 126.5, 126.5, 126.4, 125.0, 121.1, 120.6, 120.2, 120.0, 84.1, 24.9, 24.7. HRMS (ESI$^+$): m/z calcd for $C_{26}H_{23}BO_2$: 379.1869 (M)$^+$; found: 379.1858

2-phenylperylene: A mixture of pinacol ester (51 mg, 0.135 mmol) and bromobenzene (25 mg, 0.162 mmol) were placed in a 20 mL microwave vial. After addition of toluene (2 mL), 2M aqueous K_2CO_3 (0.35 mL), $Pd_2(dba)_3$ (11 mg, 0.0135 mmol), RuPhos (6.3 mg, 0.0135 mmol), ethanol (0.3 mL) and a few drops of Aliquat 336, the vial was capped and sparged with argon for 10 min. The reaction mixture was then stirred at 100 °C overnight. After cooling, the resulting mixture was diluted with water and extracted with toluene. Purification by flash chromatography using toluene as an eluent afforded the title product as a yellow solid (36 mg, 82 %). mp = 174-176 °C. λ_{max} / nm (ε / 10^4 M^{-1} cm^{-1}) 443 (3.6), 417 (2.4), 392 (1.2). v_{max}/cm^{-1} = 3053 (CH). ^{1}H NMR (400 MHz, $CDCl_3$) δ = 8.42 (dd, *J* = 1.7 Hz, 1H), 8.27 (dd, *J* = 7.6, 1.0 Hz, 1H), 8.18 (ddd, *J* = 8.9, 7.6, 1.0 Hz, 2H), 7.87 – 7.65 (m, 6H), 7.55 – 7.45 (m, 5H), 7.44 – 7.38 (m, 1H). ^{13}C NMR (101 MHz, $CDCl_3$) δ = 141.1, 139.2, 135.1, 134.8, 131.7, 131.2, 131.2, 131.1, 128.9, 128.9, 128.1, 128.1, 128.0, 127.6, 127.4, 127.0, 126.5, 126.5, 125.8, 120.4, 120.3, 120.2, 119.9. GC-MS (ES$^+$): (M)$^+$ 328.1

c) 3-phenylperylene

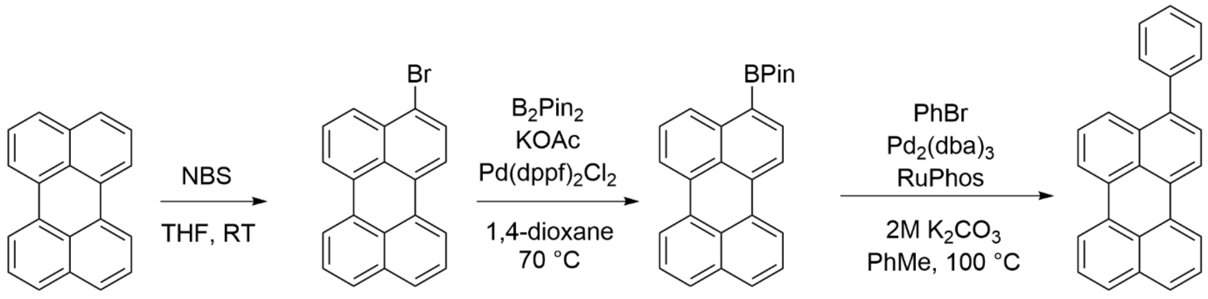

Scheme S3: Synthetic route to 3-phenylperylene

3-bromoperylene: Anhydrous THF (120 mL) was added to a round-bottom flask containing perylene (1.0 g, 3.97 mmol). After complete dissolution, *N*-bromosuccinimide (0.7 g, 3.97 mmol) in 5 mL of anhydrous THF was added. The reaction mixture was stirred at room temperature under nitrogen atmosphere overnight, after which 200 mL of water was added. The reaction mixture was extracted with dichloromethane (2 x 200 mL) and the organic phase washed with brine and dried over Na_2SO_4 and filtered. Solvents were removed under reduced pressure to afford the title compound as a yellow solid (1.25 g, 95%). ^{1}H NMR (400 MHz, $CDCl_3$): δ = 8.27-8.17 (m, 4H), 8.11 (d, J = 8.7 Hz, 1H), 8.02 (d, J = 8.4 Hz, 1H), 7.78 (d, J = 8.4 Hz, 1H), 7.73-7.67 (m, 2H), 7.62-7.57 (m, 1H), 7.53-7.47 (m, 2H). ^{13}C NMR (101 MHz, $CDCl_3$): δ = 150.0, 134.6, 133.1, 131.6, 131.2, 130.7, 130.6, 130.4, 129.8, 128.4, 128.3, 127.9, 127.8, 122.3, 121.1, 120.9, 120.6, 120.5. GC-MS (ES$^+$): m/z (M)$^+$ = 330.

3-(4,4,5,4-tetramethyl-1,3-dioxa-2-borolan-2-yl)perylene: Into a 250 mL Schlenk flask containing a mixture of 3-bromoperylene (1.0 g, 3 mmol), bis(pinacolato)-diboron (1.14 g, 4.5 mmol), Pd(dppf)$_2$Cl$_2$ (90 mg, 0.1 mmol, 5 mol%), potassium acetate (0.9 g, 9 mmol) was added anhydrous 1,4-dioxane (150 mL). The reaction mixture was purged with argon for 30 min and then stirred under an argon atmosphere at 70 ^{o}C for 17h. The reaction mixture was then allowed to reach ambient temperature. Solvent was removed by rotary evaporation and the crude residue was purified by silica gel flash chromatography (isocratic toluene elution). The title pinacol ester was obtained as an orange powder (0.90 g, 80%).^{1}H NMR (400 MHz, $CDCl_3$): δ = 8.65 (d, J = 8.4 Hz, 1H), 8.26-8.17 (m, 4H), 8.06 (d, J = 7.5 Hz, 1H), 7.96 (t, J = 8.4 Hz, 2H), 7.56-7.46 (m, 3H), 1.43 (s, 12H). ^{13}C NMR (101 MHz, $CDCl_3$): δ = 138.2, 136.1, 134.3, 133.8, 131.2, 131.0, 130.9, 130.8, 128.5, 128.3, 127.9, 127.8, 126.8, 126.7, 126.6, 120.9, 120.3, 120.2, 120.1, 119.3, 83.7, 24.9. GC-MS (ES$^+$): m/z (M)$^+$ = 378.

3-phenylperylene: A mixture of pinacol ester (150 mg, 0.4 mmol) and bromobenzene (75 mg, 0.476 mmol) were placed in a 20 mL microwave vial. After addition of toluene (6 mL), 2M aqueous K_2CO_3 (1 mL), Pd$_2$(dba)$_3$ (32.6 mg, 0.04 mmol), RuPhos (18.6 mg, 0.04 mmol), ethanol (0.8 mL) and a few drops of Aliquat 336 the vial was capped and sparged with argon for 10 min. The reaction mixture was then stirred at 100 °C overnight. After cooling, the resulting mixture was diluted with water and extracted with toluene. Purification by flash chromatography using toluene as an eluent afforded the title product as a yellow solid (120 mg, 91 %). mp = 204-206 ^{o}C. λ_{max} / nm (ε / 10^4 M^{-1} cm^{-1}) 448 (3.7), 422 (2.9), 399 (1.4). ν_{max}/cm^{-1} = 3053 (CH). ^{1}H NMR (400 MHz, $CDCl_3$) *δ* = 8.25 – 8.18 (m, 4H), 7.75 (dd, *J* = 8.4, 1.0 Hz, 1H), 7.68 (dd, *J* = 8.3, 0.9 Hz, 2H), 7.53 – 7.38 (m, 9H). ^{13}C NMR (101 MHz, $CDCl_3$) *δ* = 140.7, 140.0, 134.7, 132.9, 131.4, 131.2, 130.6, 129.9, 129.0, 128.6, 128.4, 127.8, 127.8, 127.7, 127.4, 126.6, 126.6, 126.5, 126.1, 120.3, 120.3, 120.1, 119.9. GC-MS (ES$^+$): (M)$^+$ = 328.2

7. NMR Spectra of final compounds

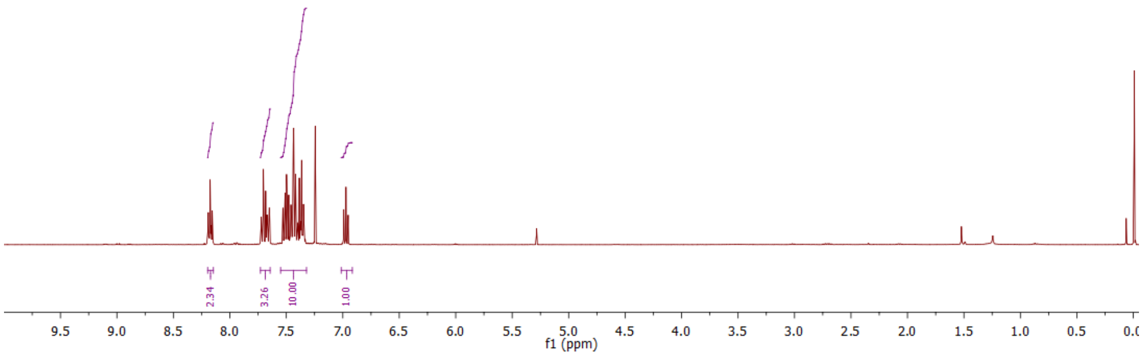

Figure S6: ^{1}H NMR of 1-phenylperylene in $CDCl_3$

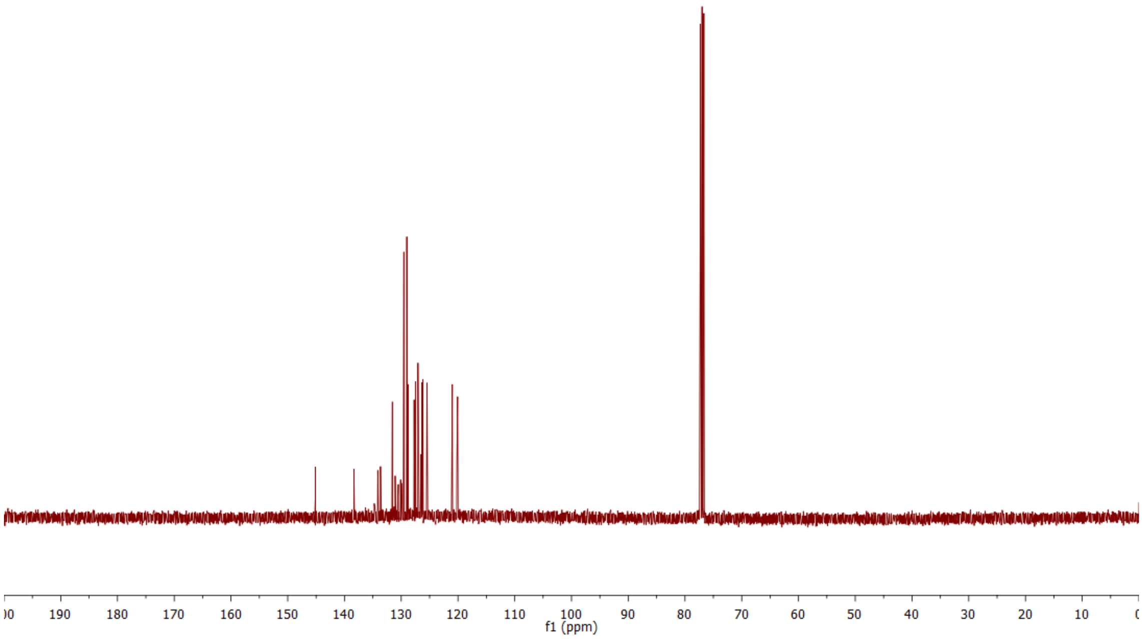

Figure S7: ^{13}C NMR of 1-phenylperylene in $CDCl_3$

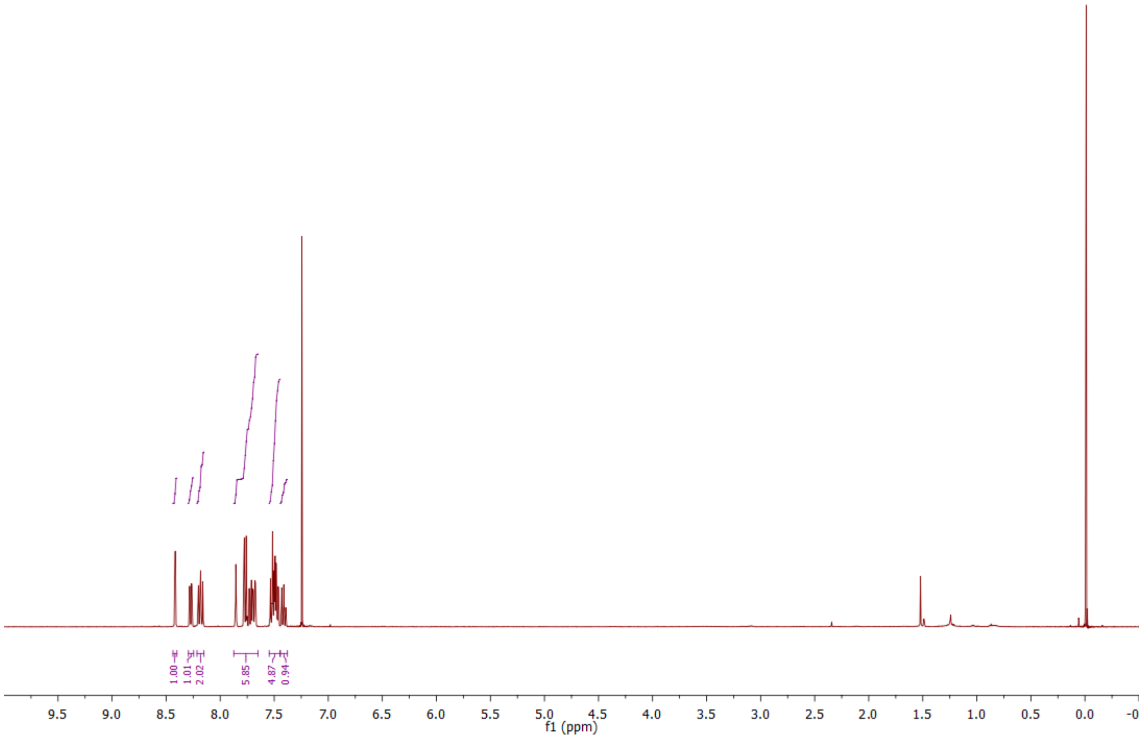

Figure S8: ^{1}H NMR of 2-phenylperylene in $CDCl_3$

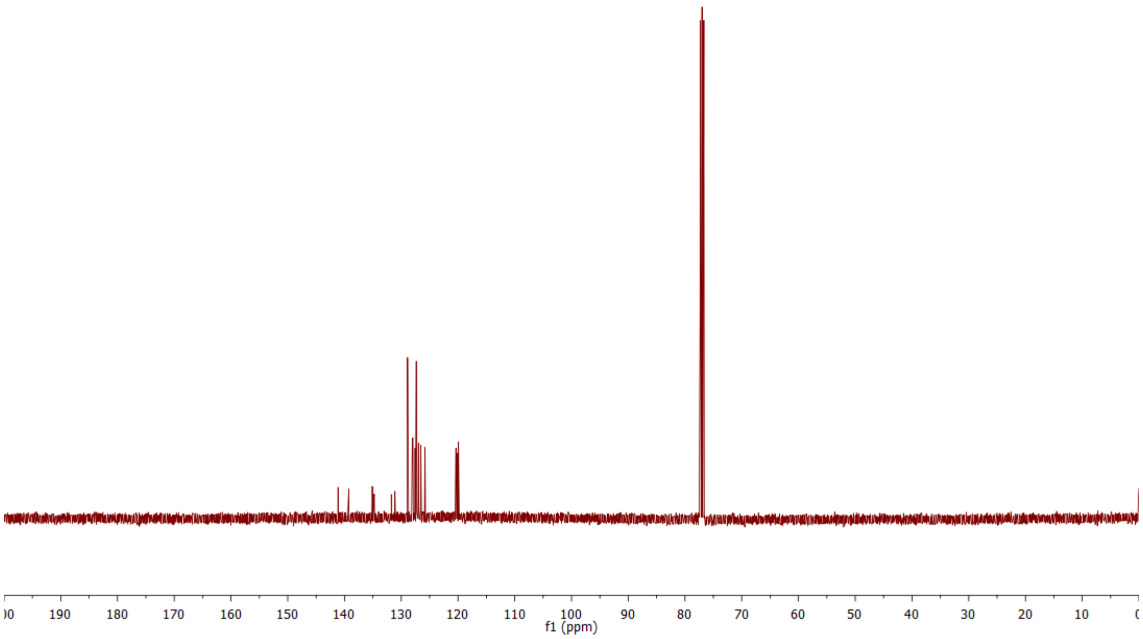

Figure S9: ^{13}C NMR of 2-phenylperylene in $CDCl_3$

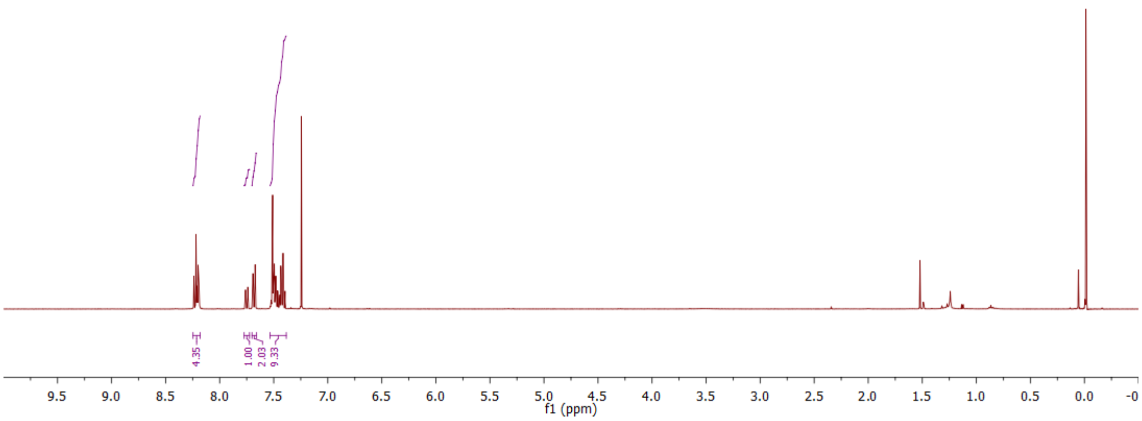

Figure S10: ^{1}H NMR of 3-phenylperylene in $CDCl_3$

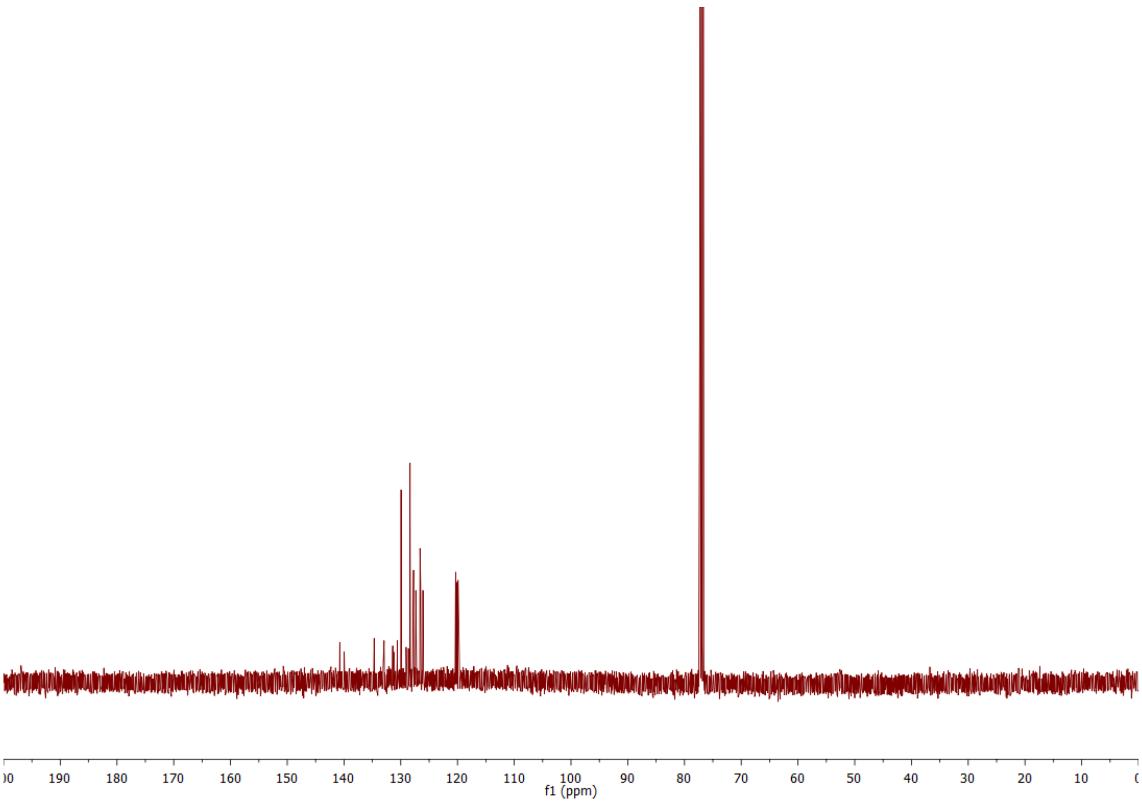

Figure S11: ^{13}C NMR of 3-phenylperylene in $CDCl_3$

8. References

1. U. Resch-Genger and K. Rurack, *Pure Appl. Chem.*, 2013, **85**, 2005-2013.
2. A. M. Brouwer, *Pure Appl. Chem.*, 2011, **83**, 2213-2228.
